# Assessment of nailfold microcirculation in non-dialysis CKD: Capillaroscopic indicators correlated with renal dysfunction

**DOI:** 10.1371/journal.pone.0359419

**Published:** 2026-09-30

**Authors:** Shulin Li, Ying Liu, Han Yan, Xinglei Zhou, Qi Liu, Yousuf Abdulkarim Waheed, Disheng Wang, Dong Sun

**Affiliations:** 1 Department of Nephrology, Affiliated Hospital of Xuzhou Medical University, Xuzhou, China; 2 Clinical Research Center for Kidney Disease Xuzhou Medical University, Xuzhou, China; 3 Department of Nephrology, Second Affiliated Hospital of Xuzhou Medical University, Xuzhou, China; 4 Department of Internal Medicine and Diagnostics, Xuzhou Medical University, Xuzhou, China; University of Pisa, ITALY

## Abstract

Chronic kidney disease (CKD) is commonly associated with vascular injury. To date, efficient tools for assessing microcirculatory changes in non-dialysis CKD patients remain limited. We conducted a retrospectively registered study to employed nailfold capillaroscopy as a non-invasive method to evaluate peripheral microcirculation in CKD. A total of 104 participants were enrolled, including 63 patients with non-dialysis CKD patients (CKD group) and 41 healthy controls (control group). Renal function assessment and nailfold microcirculation examinations were performed for all participants. We analyzed 20 microcirculatory parameters and further explored their associations with serum creatinine (Scr), blood urea nitrogen (BUN), cystatin C, estimated glomerular filtration rate (eGFR), CKD stage, and hemoglobin (Hb) within the CKD group. Results showed that serum creatinine, cystatin C, and CKD stage were independently and positively associated with the capillary number integral, whereas eGFR and hemoglobin were independently and negatively associated with the capillary number integral. In addition, serum creatinine, cystatin C, and CKD stage were independently and negatively associated with the subpapillary venular plexus integral, while eGFR was independently and positively associated with the subpapillary venular plexus integral. In summary, our findings suggest that several key renal function parameters are independently associated with capillary number integral and subpapillary venular plexus integral. These findings indicate that renal dysfunction may be associated with reduced capillary number and impaired subpapillary venous plexus relaxation in patients with non-dialysis CKD.

## Introduction

The kidneys, as highly vascularized organs, play a crucial role in regulating blood flow. Kidney dysfunction and renal damage for more than three months can be replicated as CKD [1]. The leading causes of CKD include diabetes, hypertension (HTN), primary or secondary glomerulopathies, obstructive nephropathy, medications, and genetic disorders. Among these, HTN and diabetes are of particular concern [2,3]. Most CKD patients have a vascular injury, and traditional risk factors such as hyperlipidemia and hypertension, and non-traditional ones include uremia, disordered mineral-bone disease, inflammation, and oxidative stress participate in vascular lesion [4].

Studies indicate that abnormalities in microcirculatory structure and function can impair organ function and contribute to organ damage, as seen in diabetes, HTN, and kidney disease [5–7]. HTN, uremic toxin, mineral metabolic disorders, and others can also cause vascular injury in CKD patients, resulting in vascular structure changes and dysfunction [8–10]. Microvascular rarefaction has emerged as an early marker for assessing microvascular function and tissue perfusion, including that of the kidneys [11–13]. Skin microcirculation can be deliberated as the representative of the structure and function of systemic microcirculation [14,15]. The finger nailfold is the skin wrinkle covering the root of the nail [16]. Nailfold microcirculation is the circulation pathway consisting of nailfold capillaries, a typical peripheral circulation pathway. To a certain extent, nailfold capillaries can reflect changes of microcirculatory status and can be utilized as a window to evaluate microcirculatory status. To date, nailfold microcirculation research has primarily focused on rheumatic and immune diseases, with limited attention to kidney disease. Previous studies have demonstrated altered nailfold microcirculation in CKD patients undergoing dialysis, with a strong association between these changes and dialysis treatment [17,18].

The present study aimed to explore the relationship between nailfold capillary indexes and renal function, and to develop a novel, non-invasive, and efficient technique for assessing the microcirculatory status of non-dialysis CKD patients.

## Methods

### Ethical approval

The study was conducted at the Xuzhou Medical University Affiliated Hospital. Our study has been registered at Clinical Trials. gov (NCT03682952) and has been approved by the Clinical Trial Ethics Committee of Xuzhou Medical University Affiliated Hospital (XYFY2018-KL034–01) Helsinki and its later amendments or comparable ethical standards. All participants provided written informed consent prior to participation.

### Subjects

We recruited 41 healthy individuals into the control group from Physical Examination Center of the Affiliated Hospital of Xuzhou Medical University. We recruited 68 non-dialysis CKD patients into the CKD group who accepted treatment in the Department of Nephrology of the Affiliated Hospital of Xuzhou Medical University. Following ethics committee approval, data were accessed for research purposes between 01/10/2018 and 31/12/2020. We had strict exclusion and inclusion criteria for CKD patients. Inclusion criteria: non-dialysis CKD patients with strict blood pressure control (systolic blood pressure ≤ 140 mmHg, diastolic blood pressure ≤ 90 mmHg). Exclusion criteria: Combined with other diseases that can affect systemic blood circulation and vascular function, including cardiovascular, cerebrovascular, respiratory, endocrine, rheumatic immune diseases, infection, shock, dehydration, urgent dialysis, and other emergencies. Among the CKD group, 5 CKD patients were excluded from the group. Those are as follows: blood pressure of 2 CKD patients did not reach up the criteria, nailfold microcirculation images of 2 CKD patients were affected by nailfold skin injury, and nailfold microcirculation result of 1 CKD patient was affected by the thickness of nailfold cutin. They were excluded from the CKD group. The remaining 63 CKD patients were included in the CKD group (Fig 1). We collected the medical history of the patients together with basic information. All members accepted hematological examination, including Scr, BUN, cystatin C, Hb. Also, we calculated eGFR according to the CKD-EPI formula. The detailed information regarding CKD etiology and the distribution of CKD stages are now presented in Tables 1 and 2.

**Fig 1:**
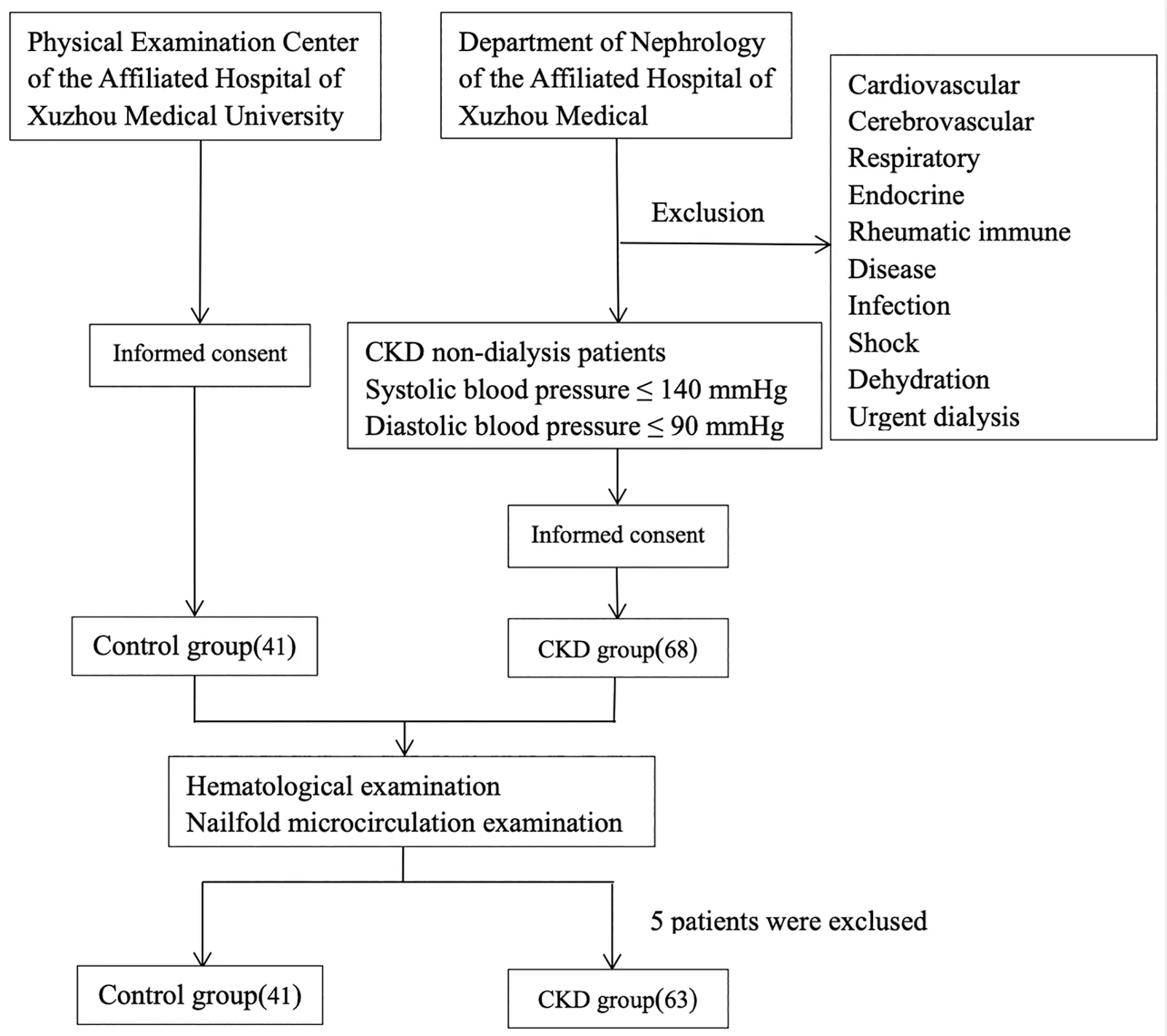
We recruited 41 healthy individuals from Physical Examination Center of the Affiliated Hospital of Xuzhou Medical University, then we informed them of the experimental procedure, and after their informed consent, they were included in the control group. We recruited non-dialysis CKD patients who accepted treatment in the Department of Nephrology of the Affiliated Hospital of Xuzhou Medical University, we exculuded the patients who were combined with other diseases that can affect systemic blood circulation and vascular function, including cardiovascular, cerebrovascular, respiratory, endocrine, rheumatic immune diseases, infection, shock, dehydration, urgent dialysis, and other emergencies, 68 non-dialysis CKD patients were included in the CKD group after informed consent, after relevant examinations, 5 patients were excluded, and ultimately 63 non-dialysis CKD patients were included in the CKD group.

### Nailfold microcirculation examination

The nailfold microcirculation examination is a rapid, non-invasive, and quantitative technique to evaluate the nailfold capillary status using nailfold capillaroscopy. Before the examination, subjects were required to sit in a room for 15–20 minutes calm, their hands were needed to be warm, and the nailfold was uninjured, ensuring that we could clearly observe capillaries and blood flow. In the examination, pine and cypress oil were dripped on the skin of fourth finger nailfold. We evaluated morphology of capillaries, blood flow, and pericapillary state from the image (Fig 2a) pictured by nailfold capillaroscopy, which obtained 20 parameterized indexes. The 20 parameterized indexes such as clarity integral, capillary number integral, input branch diameter integral, output branch diameter integral, output branch diameter/input branch diameter, capillary top diameter integral, capillary length integral, crossed capillary number integral, abnormal capillary number integral, the velocity of blood flow integral, vascular motility integral, erythrocyte aggregation integral, white blood cell number integral, white microthrombus integral, the color of blood integral, perivascular exudation integral, capillary hemorrhage integral, subpapillary venular plexus integral, nailfold nipple integral and sweat gland catheter integral(Table 1). Our results showed that the image of nailfold microcirculation was clear, the nail fold capillaries were wavy, the capillary shape was normal, and the blood flow was good in the control group. However, with the progression of CKD, the image of nailfold microcirculation was blurred, the number of capillaries was reduced, the blood flow was poor, and in severe cases, the nail fold papillae were flat and the capillary morphology was abnormal in the CKD group (Fig 2b).

**Table 1:** 20 indexes of nailfold microcirculation examination.

Index	Normal state	Comprehensive judgment
clarity integral	image is clear	the sate of nailfold microcirculation was abnormal, the corresponding integral increased, resulting in abnormal comprehensive judgment.
capillary number integral	capillary number is 7-9 strips/mm	
input branch diameter integral	input branch diameter is 9-13 um	
output branch diameter integral	input branch diameter is 11-17 um	
output/input branch diameter	1.3	
capillary top diameter integral	capillary top diameter is 12-18 um	
capillary length integral	capillary top diameter is 150-250 um	
crossed capillary number integral	crossed capillary number<30%	
abnormal capillary number integral	crossed capillary number<10%	
velocity of blood flow integral	velocity of blood flow>600um/s	
vascular motility integral	0-1 time/minute	
erythrocyte aggregation integral	there is no erythrocyte aggregation	
white blood cell number integral	white blood cell number is 1-30/15s	
white microthrombus integral	There is no white microthrombus	
color of blood integral	white blood cell number is light red	
perivascular exudation integral	there is no perivascular exudation	
capillary hemorrhage integral	there is no capillary hemorrhag	
subpapillary venular plexus integral	there is no subpapillary venular plexus	
nailfold nipple integral	nailfold nipple is corrugated	
sweat gland catheter integral	sweat gland catheter is 0-2/nailfold	

**Fig 2:**
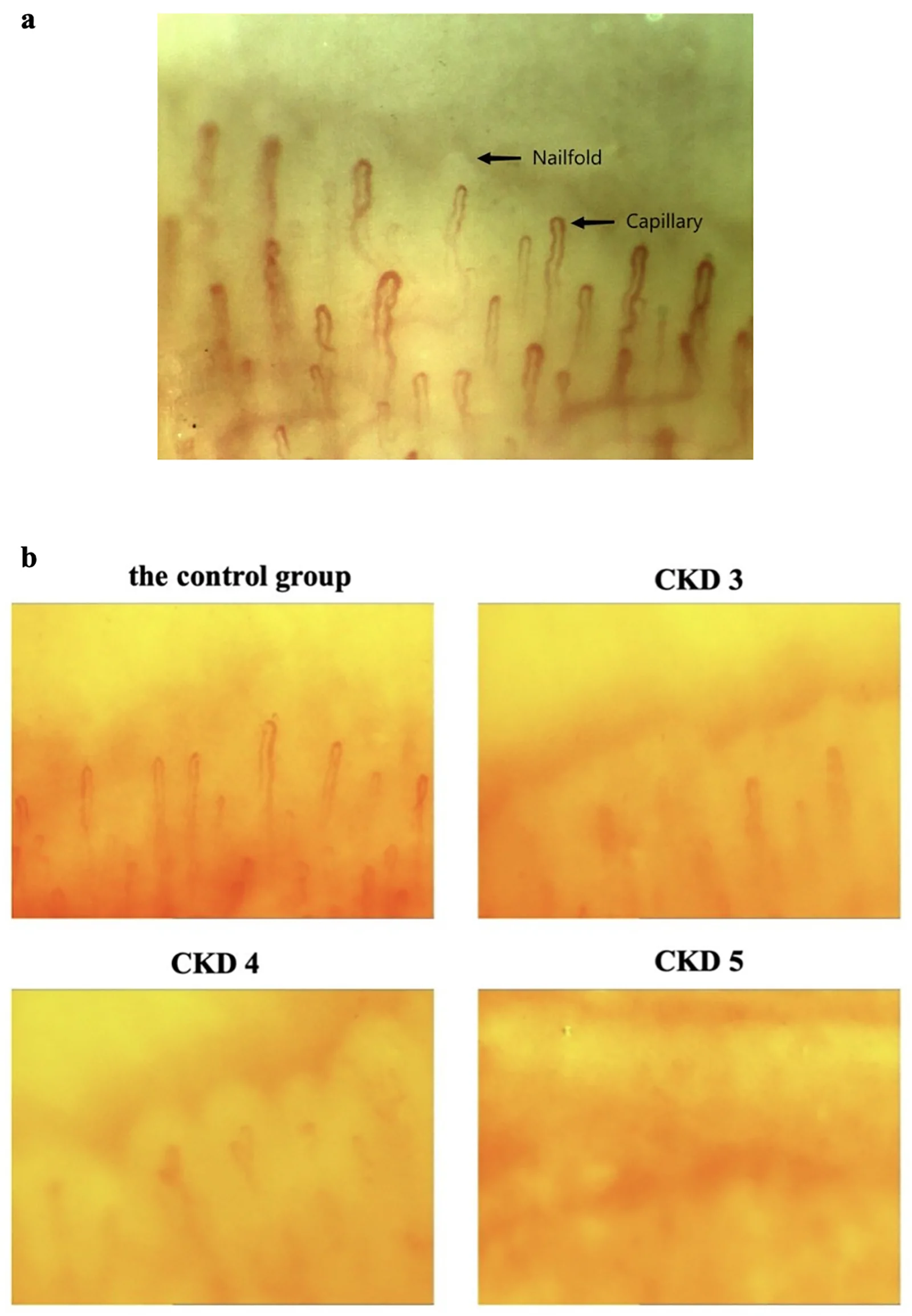
(a) Image of nailfold microcirculation pictured by nailfold capillary scopy. (b) In the control group, the image of nailfold microcirculation was clear, the nail fold capillaries were wavy, the capillary shape was normal, and the blood flow was good. In CKD group, with the progression of CKD, the image of nailfold microcirculation is blurred, the number of capillaries is reduced, the blood flow is poor, and in severe cases, the nail fold papillae are flat and the capillary morphology is abnormal.

Table 1 Nailfold microcirculation examination obtained 20 parameterized indexes, if the 20 parameterized indexes were not normal, the corresponding integral increased, resulting in abnormal comprehensive judgment.

### Nailfold microcirculation examination analysis methods

We applied the Niu Tian integral method to assess and grade the status of nailfold microcirculation. According to the changes of the microcirculation indexes and weighted integral values, the comprehensive judgment can be divided into 5 degrees. The total integral is greater than or equal to 8, or one of the following changes occured, the comprehensive judgment is severely abnormal: a. the capillary number was reduced to 3 strips/mm, or the capillary number decreased by more than 80%; b. red blood cells accumulated heavily, the blood flow was slow or even completely stopped; c. most white microemboli in the blood flow were caused by non-local factors. d. in one nailfold, the number of capillary hemorrhage was greater than or equal to 7. The total integral is greater than or equal to 4, or two of the following changes occured, the comprehensive judgment is moderately abnormal: a. the capillary number decreased by 40% ~ 60%; b. the input branch diameter reduced by 20% to 60% or widened by 60%; c. the capillary length shortened by 80% or increased by more than 50%; d. the output branch diameter widened by more than 100%; e. perivascular exudation was obvious; f. most capillary blood flow is granular; g. in one nailfold, the number of capillary hemorrhage up to 3–6 strips; h. the color of blood was dark red; i. morphology of capillaries changed in a short time, the number of deformed and crossed capillaries up to 40%−60%; j. dermal papillae of nailfold is flat. The total integral is greater than 2, or two of the following changes occured, the comprehensive judgment is the comprehensive judgment is mildly abnormal: a. the input branch diameter, output branch diameter or capillary top diameter widened or narrowed up to 20%; b. the capillary length increased by 25%−50% or shortened by 20%; c. the number of deformed and crossed capillaries up to 40%; d. the blood flow is grained; e. in one nailfold, the number of capillary hemorrhage or perivascular exudation up to 1–2 strips; f. subpapillary venular plexus is obvious, dilated and thickened; g. the capillary is vague; h. the number of white blood cell is greater than 30 per 15 seconds or there is no white blood cell in blood flow; i. the number of sweat gland catheter in a nailfold is 3–4. The total integral is less than or equal to 2, the nailfold microcirculation is almost normal. The total integral is less than 1, the nailfold microcirculation is normal(Table 2).

**Table 2:** The comprehensive judgment of nailfold microcirculation examination.

Comprehensive judgment (5 degrees)	Abnormal changes of nailfold microcirculation
**severely abnormal** the total integral is greater than or equal to 8, or one of the abnormalchanges occured within the right-hand column (changes of nailfold microcirculation)	a. the capillary number was reduced to 3 strips/mm, or the capillary number decreased by more than 80%; b. red blood cells accumulated heavily, the blood flow was slow or even completely stopped; c. most white microemboli in the blood flow were caused by non-local factors; d. in one nailfold, the number of capillary hemorrhage was greater than or equal to 7.
**moderately abnormal** the total integral is greater than or equal to 4, or two of the abnormalchanges occured within the right-hand column (changes of nailfold microcirculation)	a. the capillary number decreased by 40%−60%; b. the input branch diameter reduced by 20% to 60% or widened by 60%; c. the capillary length shortened by 80% or increased by more than 50%; d. the output branch diameter widened by more than 100%; e. perivascular exudation was obvious; f. most capillary blood flow is granular; g. in one nailfold, the number of capillary hemorrhage up to 3–6 strips; h. the color of blood was dark red; i. morphology of capillaries changed in a short time, the number of deformed and crossed capillaries up to 40%−60%; j. dermal papillae of nailfold is flat.
**mildly abnormal** the total integral is greater than 2, or two of the abnorma changes occured within the right-hand column (changes of nailfold microcirculation)	a. the input branch diameter, output branch diameter or capillary top diameter widened or narrowed up to 20%; b. the capillary length increased by 25%−50% or shortened by 20%; c. the number of deformed and crossed capillaries up to 40%; d. the blood flow is grained; e. in one nailfold, the number of capillary hemorrhage or perivascular exudation up to 1–2 strips; f. subpapillary venular plexus is obvious, dilated and thickened; g. the capillary is vague; h. the number of white blood cell is greater than 30 per 15 seconds or there is no white blood cell in blood flow; i. the number of sweat gland catheter in a nailfold is 3–4
**almost normal**	the total integral is less than or equal to 2
**normal**	the total integral is less than 1

Table 2 The comprehensive judgment of nailfold microcirculation examination can be divided into 5 degrees, if the total integral reached a certain level or there were abnormal changes in nail fold microcirculation, it directly affected the comprehensive judgment.

### Statistical analysis

SPSS 22.0 statistical software was used to analyze the data. Under the normal distribution, the data were expressed by the mean ± standard deviation (x̄±s). Independent-samples t tests were used to compare the age, systolic pressure, diastolic pressure, cystatin C and Hb between the two groups. The data that do not accord with the normal distribution is expressed by the median (quartile). U tests were used to compare the Scr, BUN, and eGFR. The chi-square test was used to compare sex between the two groups. The correlations between Scr, BUN, eGFR, cystatin C, Hb, CKD stage, and meaningful indexes of nailfold microcirculation were analyzed by Spearman correlation analysis. Furthermore, adjusted multiple linear regression analyses were performed to evaluate the independent associations between renal function parameters and nailfold microcirculatory abnormalities. Separate models were constructed using the capillary number integral and subpapillary venular plexus integral as the dependent variables. Renal function parameters were entered as independent variables, with age, blood pressure, hemoglobin level, and CKD etiology included as covariates to adjust for potential confounding factors. *P* < 0.05 indicates a statistically significant difference.

## Results

### General data

We conducted statistical analysis on the sex, age, systolic blood pressure and diastolic blood pressure of CKD group and control group. No significant differences were observed between the two groups for these variables (Table 3, *P* > 0.05 for all).

**Table 3:** Comparison of general data between the two groups.

Index	Control group (n = 41)	CKD group (n = 63)	*t/χ2/Z*	*P*
male	20 (48.8)	34 (54.0)	0.268	0.605
female	21 (51.2)	29 (46.0)		
age	43.12 ± 13.70	46.19 ± 12.41	1.183	0.240
systolic blood pressure	126.54 ± 6.02	128.21 ± 9.88	0.971	0.334
diastolic blood pressure	80.00 ± 4.68	80.03 ± 6.70	0.026	0.979
Scr (umol/L)	60.00 (55.00,70.00)	224.00 (120.00,390.00)	−8.463	<0.001
BUN (mmol/L)	4.40 (3.80,5.50)	14.80 (7.44,1.10)	−8.190	<0.001
eGFR (ml/min/1.73m^2^)	111.21 (102.07,119.03)	23.21 (12.34,56.84)	−8.591	<0.001
cystatin C (mg/L)	0.61 ± 0.09	2.07 ± 0.95	9.797	<0.001
Hb (g/L)	138.24 ± 13.19	113.30 ± 26.25	5.633	<0.001

Table 3 Sex, age, systolic blood pressure, and diastolic blood pressure were not significantly different between the control group and the CKD group. While Scr, BUN and cystatin C, eGFR and Hb were different between the control group and the CKD group. Scr, BUN and cystatin C of the control group were significantly lower than in CKD group, whereas eGFR and Hb were increased in the control group (*P* < 0.05).

### Hematological indexes

We conducted statistical analysis on the scr, BUN, cystatin C, eGFR and Hb of CKD group and control group. Scr, BUN and cystatin C of the control group were significantly lower than in CKD group, whereas eGFR and Hb were increased in the control group(Table 3, *P* < 0.001 for all).

### Nailfold microcirculation examination

Nailfold microcirculation examination has 20 parameterized indexes, we also conducted statistical analysis on the 20 parameterized indexes of CKD group and control group. Compared with the control group, we found that the capillary number integral, output branch diameter integral, capillary top diameter integral, crossed capillary number integral, the velocity of blood flow integral, erythrocyte aggregation integral, perivascular exudation integral, and capillary hemorrhage integral were increased in the CKD group, whereas the capillary length integral color of blood integral, and subpapillary venular plexus integral were decreased in the CKD group (Table 4, *P* < 0.05 for all).

**Table 4:** Comparison of nailfold microcirculation examination 20 indexes between the two groups.

Index	Control group (n = 41)	CKD group (n = 63)	*Z*	*P*
clarity integral	0.00 (0.00, 0.20)	0.00 (0.00, 0.00)	−1.565	0.118
capillary number integral	0.80 (0.40, 0.80)	2.00 (0.80, 2.00)	−3.949	<0.001
input branch diameter integral	0.00 (0.00, 0.20)	0.20 (0.00, 0.80)	−1.788	0.074
output branch diameter integral	0.00 (0.00, 0.00)	0.00 (0.00, 0.40)	−2.871	0.004
output/input branch diameter	1.20 (1.00, 1.30)	1.30 (1.00, 1.30)	−0.375	0.708
capillary top diameter integral	0.00 (0.00, 0.10)	0.10 (0.00, 1.00)	−2.465	0.014
capillary length integral	0.20 (0.00, 0.20)	0.00 (0.00, 0.20)	−2.618	0.009
crossed capillary number integral	0.00 (0.00, 0.00)	0.00 (0.00, 0.20)	−1.970	0.049
abnormal capillary number integral	0.00 (0.00, 0.00)	0.00 (0.00, 0.20)	−1.558	0.119
velocity of blood flow integral	0.00 (0.00, 0.00)	0.40 (0.00, 0.40)	−4.414	<0.001
vascular motility integral	0.00 (0.00, 0.00)	0.00 (0.00, 0.00)	−1.754	0.079
erythrocyte aggregation integral	0.00 (0.00, 0.00)	0.20 (0.00, 0.20)	−3.864	<0.001
white blood cell number integral	0.00 (0.00, 0.00)	0.00 (0.00, 0.00)	−1.762	0.078
white microthrombus integral	0.00 (0.00, 0.00)	0.00 (0.00, 0.00)	−0.308	0.758
color of blood integral	0.00 (0.00, 0.00)	0.00 (0.00, 0.00)	−2.088	0.037
perivascular exudation integral	0.00 (0.00, 0.00)	0.00 (0.00, 1.50)	−3.742	<0.001
capillary hemorrhage integral	0.00 (0.00, 0.00)	0.80 (0.00, 0.80)	−4.297	<0.001
subpapillary venular plexus integral	0.00 (0.00, 0.10)	0.00 (0.00, 0.00)	−2.597	0.009
nailfold nipple integral	0.00 (0.00, 0.40)	0.00 (0.00, 0.40)	−1.163	0.245
sweat gland catheter integral	0.00 (0.00, 0.00)	0.00 (0.00, 0.00)	−1.240	0.215

*Indexes of nailfold microcirculation examination mentioned above named meaningful indexes.

Table 4 20 parameterized indexes of nailfold microcirculation examination were different between the control group and the CKD group. The capillary number integral, output branch diameter integral, capillary top diameter integral, crossed capillary number integral, the velocity of blood flow integral, erythrocyte aggregation integral, perivascular exudation integral, and capillary hemorrhage integral were increased in the CKD group (*P* < 0.05), whereas the capillary length integral color of blood integral, and subpapillary venular plexus integral were decreased in the CKD group (*P* < 0.05).

### Correlation between renal function indexes and meaningful indexes in the CKD group

Based on the above results, we conducted statistical analysis on the correlation between renal function indexes and meaningful indexes in the CKD group. Scr, BUN, cystatin C, CKD stage had positive correlation with capillary number intergral(Table 5, *P* < 0.05 for all) (Fig 3a-d); eGFR had negative correlation with capillary number intergral(Table 5, *P* < 0.05) (Fig 3e); Scr, cystatin C, CKD stage had negative correlation with subpapillary venular plexus intergral(Table 5, *P* < 0.05 for all) (Fig 3f-h); eGFR had positive correlation with subpapillary venular plexus intergral(Table 5, *P* < 0.05) (Fig 3i).

**Table 5:** Correlation between capillary number integral, subpapillary venular plexus integral, and renal function indexes in the CKD group.

Index	Capillary number integral		Subpapillary venular plexus	
	*r*_s_	*P*	*r*_s_	*P*
Scr (umol/L)	0.348	0.005	−0.310	0.013
BUN (mmol/L)	0.332	0.008	−0.234	0.065
eGFR (ml/min/1.73m^2^)	−0.401	0.001	0.310	0.013
cystatin C (mg/L)	0.303	0.016	−0.297	0.018
CKD stage	0.398	0.001	−0.308	0.014
Hb (g/L)	−0.441	<0.001	0.121	0.334

**Fig 3:**
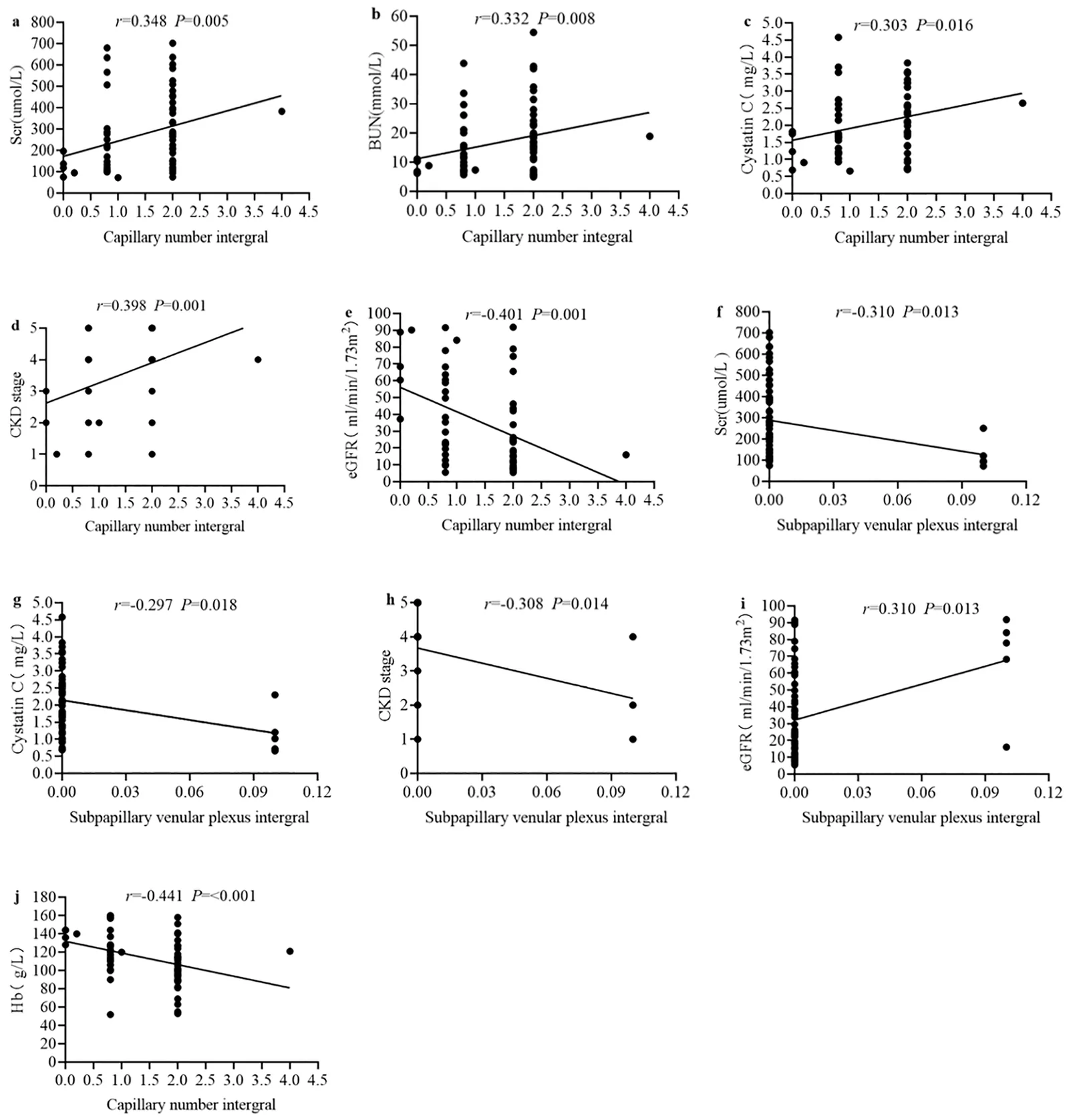
(a) Scr has a positive correlation with the capillary number integral. (b) BUN has a positive correlation with the capillary number integral. (c) Cystatin C has a positive correlation with the capillary number integral. (d) The CKD stage has a positive correlation with the capillary number integral. (e) eGFR has a negative correlation with the capillary number integral. (f) Scr has a negative correlation with subpapillary venular plexus integral. (g) Cystatin C has a negative correlation with subpapillary venular plexus integral. (h) The CKD stage has a negative correlation with subpapillary venular plexus integral. (i) eGFR has a positive correlation with subpapillary venular plexus integral. (j) Hb has a negative correlation with the capillary number integral.

Table 5 Hematological indexes have connection with capillary number integral, subpapillary venular plexus integral. Scr, BUN, cystatin C, CKD stage had positive correlation with capillary number intergral (*P* < 0.05), while Scr, cystatin C, CKD stage had negative correlation with subpapillary venular plexus intergral (*P* < 0.05); eGFR, Hb had negative correlation with capillary number intergral (*P* < 0.05), while eGFR had positive correlation with subpapillary venular plexus intergral (*P* < 0.05).

### Correlation between Hb and meaningful indexes of in the CKD group

We conducted statistical analysis on the correlation between Hb and meaningful indexes in the CKD group too. Hb showed a negative correlation with the capillary number integral (Table 3, *P* < 0.05) (Fig 3j) and positive correlations with the capillary top diameter integral and blood flow color integral (*P* = 0.047).

### Adjusted multivariable linear regression analysis

Adjusted multivariable linear regression analyses were performed to evaluate the independent associations between renal function parameters and nailfold microcirculatory abnormalities after controlling for potential confounding factors. Age, blood pressure, hemoglobin level, and CKD etiology were included as covariates in the regression models. Serum creatinine, cystatin C, and CKD stage remained independently and positively associated with the capillary number intergral, whereas eGFR and hemoglobin remained independently and negatively associated with the capillary number intergral. Similarly, serum creatinine, cystatin C, and CKD stage remained independently and negatively associated with the subpapillary venular plexus intergral, while eGFR remained independently and positively associated with the subpapillary venular plexus intergral (Tables 6 and 7).

**Table 6:** Independent associations of capillary number integral with renal function parameters.

Index	Capillary number intergral			
	Univariate Spearman’s *r*_s_	Adjusted standardized β	*P* value	Direction of independent association
Scr (μmol/L)	0.348	0.287	0.012	Positive independent association
BUN (mmol/L)	0.332	0.156	0.137	No independent association
eGFR (ml/min/1.73m²)	−0.401	−0.364	<0.001	Negative independent association
Cystatin C (mg/L)	0.303	0.251	0.021	Positive independent association
CKD stage	0.398	0.326	0.003	Positive independent association
Hb (g/L)	−0.441	−0.312	0.005	Negative independent association

**Table 7:** Independent associations of subpapillary venular plexus intergral with renal function parameters.

Index	Subpapillary venular plexus intergral			
	Univariate Spearman’s *r*_s_	Adjusted standardized β	*P* value	Direction of independent association
Scr (μmol/L)	−0.310	−0.263	0.018	Negative independent association
BUN (mmol/L)	−0.234	−0.115	0.272	No independent association
eGFR (ml/min/1.73m²)	0.310	0.331	0.002	Positive independent association
Cystatin C (mg/L)	−0.297	−0.240	0.026	Negative independent association
CKD stage	−0.308	−0.305	0.007	Negative independent association
Hb (g/L)	0.121	0.104	0.334	No independent association

## Discussion

This study mainly used nailfold capillaroscopy to investigate and evaluate various nailfold microcirculation indexes comprehensively. During the investigation, the present study suggested three findings. First, compared with healthy controls, patients with non-dialysis CKD exhibited significant nailfold microcirculatory abnormalities. Second, after adjustment for major clinical confounders, several key renal function parameters remained independently associated with nailfold microcirculatory abnormalities. Third, these findings support an independent association between renal dysfunction and nailfold microcirculatory impairment in non-dialysis CKD, although the cross-sectional design precludes any inference of causality.

Based on the data, we found that there are differences in most indexes between CKD group and control group. In CKD group, capillary number integral, output branch diameter integral, capillary top diameter integral, crossed capillary number integral, the velocity of blood flow integral, erythrocyte aggregation integral, perivascular exudation integral, and capillary hemorrhage integral were significantly higher than those in the control group. While the length integral, blood color integral, and subpapillary venular plexus integral of the CKD group were significantly lower than those in the control group. It exhibits that the alterations and changes of nailfold microcirculation were significantly different between both groups. These alterations were more severe in non-dialysis CKD patients compared with healthy individuals. Based on these findings, we hypothesize that the alterations and changes of nailfold microcirculation are strongly related to the chronic pathological process of a microvessel, which may be related to vascular damage, internal environment disturbance, hemodynamics changes, blood volume.

Alongside these investigations, we were also able to analyze the correlation between the nailfold microcirculation indexes and renal function indexes of the CKD group. The results showed that Scr, BUN, cystatin C and CKD stage had a positive correlation with capillary number integral. In contrast, the eGFR and Hb had a negative correlation with the capillary number integral. The Scr, cystatin C and CKD stage had a negative correlation with subpapillary venular plexus integral, while eGFR had a positive correlation. So with the deterioration of renal function, the number of nailfold capillaries was decreased. To further account for potential confounding factors, we performed adjusted multivariable linear regression analyses by including age, blood pressure, hemoglobin level, and CKD etiology as covariates. After adjustment, Scr, cystatin C, and CKD stage remained independently and positively associated with the capillary number intergral, whereas eGFR and hemoglobin remained independently and negatively associated with the capillary number intergral. Regarding the subpapillary venular plexus intergral, Scr, cystatin C, and CKD stage remained independently and negatively associated, while eGFR remained independently and positively associated. In contrast, blood urea nitrogen was no longer independently associated with either microcirculatory parameter after adjustment, and hemoglobin was not independently associated with the subpapillary venular plexus intergral These findings suggest that the associations between renal dysfunction and nailfold microcirculatory abnormalities are largely independent of major clinical confounding factors, thereby strengthening the robustness of our findings. Anemia led to the reduction of nailfold capillaries number too. Thang et al. discovered that the characteristic changes in skin capillaries of patients with advanced CKD are density reduction and dysfunction [19]. Furthermore, Edwards-Richards et al. revealed that nailfold capillary density of young CKD patients with hemodialysis was significantly decreased [20]. These were consistent with our finding. We know that the uremic state itself can lead to vascular endothelial growth factor production reduced, further affecting vascular regeneration, and repairment [21]. This may be related to the decrease of nailfold capillaries. Stroke is one of the death causes in CKD patients; in addition to that, CKD also has high cardiovascular morbidity and mortality [22–25]. This also indicated that the vascular condition of CKD patients was poor. Capillary rarefaction means two aspects, including structure and function, the structural aspect implies the reduction of capillary number, the functional aspect refers to the dysfunction of the capillary [11–13]. We also found that the deterioration of renal function caused the diastolic dysfunction of the subpapillary venular plexus. Stewart et al. mentioned that the microvascular results measured by non-invasive laser Doppler flowmetry correlate with coronary artery disease confirmed by coronary angiography [26]. This indicated that non-invasive microvascular examination can serve as a basis for assessing vascular sclerosis. CKD renin-angiotensin system- mediated angiotensin – II not only induces inflammatory factors but also leads to vascular fibrosis [27]. Endothelial cells are the primary barrier between blood and tissue whose structural damage and dysfunction are the beginning of vascular injury [28]. With CKD progression, the inflammation gradually increases, leading to endothelial dysfunction and ultimately causing vascular damage [29]. Oxidative stress can lead to atherosclerosis and cardiovascular time, and was also one of the main factors that damage blood vessels [30]. The above factors were present in the CKD, which may be related to the dysfunction of the subpapillary venular plexus.

We also found that Hb had negative correlation with capillary number intergral, Hb had positive correlation with capillary top diameter intergral and blood flow color intergral. The oxygen supplied to tissues or organs is related to the oxygen-carrying capacity of the blood. Reduced Hb levels lead to decreased blood oxygen content, impairing oxygen delivery to tissues. In end-stage renal disease, anemia significantly impacts blood flow and volume, altering cardiovascular structure and function [31–33]. In hypoxia, Hb can regulate blood flow by releasing vasodilators nitric oxide and adenosine triphosphate [34–36]. Hypoxia plays a vital role in physiological and pathological angiogenesis, which mainly promotes vascular growth factors [37–39]. However, in our study, the anemia caused a reduction of the nailfold capillary numbers in the CKD group.

### Strengths and limitation

This study offers several key strengths. First, it utilizes nailfold capillaroscopy—a non-invasive, rapid, and repeatable technique—to assess peripheral microcirculation, making it well-suited for clinical use. Second, the quantitative analysis of 20 microcirculation indexes provides a detailed and objective evaluation of capillary alterations in CKD. Third, several key renal function parameters were found to be associated with selected nailfold microcirculatory indices, providing further evidence of the relationship between renal dysfunction and microcirculatory abnormalities in patients with non-dialysis CKD. Our findings suggest that nailfold capillaroscopy may serve as a promising tool for investigating microvascular abnormalities in patients with non-dialysis CKD. Further studies with larger sample sizes are needed to validate these findings and explore their clinical implications.

Although we adjusted for major clinical confounding factors, residual confounding from unmeasured variables cannot be completely excluded. Because antihypertensive treatment regimens varied among patients and detailed information regarding medication dosage and treatment duration was unavailable, the potential effects of different antihypertensive medications on nailfold microcirculation could not be fully adjusted for. In addition, our sample size was relatively small, which may limit the generalizability of the findings. Future prospective studies with larger cohorts and more comprehensive assessment of medication exposure are warranted to further validate these findings and clarify the clinical significance of nailfold microcirculatory abnormalities in patients with CKD.

## Conclusion

To sum up our work, the study found that the nailfold microcirculation of non-dialysis CKD patients was altered and changed compared with healthy individuals. Several key renal function parameters were independently associated with nailfold capillary number and subpapillary venous plexus abnormalities in patients with non-dialysis CKD. These findings support an association between renal dysfunction and nailfold microcirculatory impairment. As a non-invasive, rapid, and quantitative technique, nailfold capillaroscopy may have potential research value for the rapid assessment of microvascular abnormalities in patients with non-dialysis CKD. Further studies with larger sample sizes are warranted to validate these findings.

## Abbreviations

CKD: Chronic kidney disease
Scr: serum creatinine
BUN: blood urea nitrogen
eGFR: estimated glomerular filtration rate
Hb: hemoglobin
HTN: hypertension
RAAS: renin-angiotension-aldosterone system
